# Representing Chronic Pain Experiences Through Manual and AI-Driven Vignettes From Video Blog Data With Member Checking: Participatory Design Study

**DOI:** 10.2196/80998

**Published:** 2026-09-01

**Authors:** Shehnaz Fatima Lakha, Peter Pennefather, Alamgir Khandwala, Deborah I Fels

**Affiliations:** 1 Inclusive Media and Design Centre, Ted Rogers School of Managment, Toronto Metropolitan University, 55 Dundas Street W, Toronto, ON, M5G 2C3, Canada, 1 416-979-5000 ext 7110; 2 Wellness and Health Enhancement Engineering Lab (WHEEL), Mechanical and Industrial Engineering, University of Toronto, Toronto, ON, Canada; 3 Leslie Dan Faculty of Pharmacy, University of Toronto, Toronto, ON, Canada; 4 School of Administrative Studies, York University, Toronto, ON, Canada

**Keywords:** vignettes, video blog, GenAI or ChatGPT, patient-reported outcomes, patient narratives

## Abstract

**Background:**

Health care professional–generated vignettes are commonly used to illustrate and analyze patient experiences, shaped through clinical reflection to support provider understanding, training, and practice improvement. However, the subjective interpretation of narratives and the time-consuming manual process limit this approach. As an alternative, patients could record video blogs (vlogs) of their experiences, which can then be transformed into vignettes using GenAI and carefully designed prompts.

**Objective:**

This study aims to compare the feasibility and utility of 2 different vignette creation methods, manual and ChatGPT-3.0, from user-generated vlogs about living with chronic pain.

**Methods:**

Short commentary videos were recorded by a person living with chronic pain of unknown origin. These were transcribed and used to create vignettes. From 64 videos, three 1-minute clips were selected, each representing a key life stream: university student, international traveler, and patient with chronic pain, based on prior thematic analysis. In total, 3 vignettes were created for each creation method (manual and AI-generated). Each vignette incorporated a profile description, 3 selected user-generated video clips representing the participant’s 3 life streams, researcher-generated analytic commentary for each clip (produced by a researcher with 15 years of experience in manual vignette creation for clinical use), and a brief summary of each video. A comparison between manually constructed and AI-generated vignettes evaluated coherence, contextual accuracy, narrative depth, and utility in health communication and user experience research. Additionally, the person responsible for generating the raw videos (Participant X) provided commentary on this process as a cocreator of a new method, offering critical insights into the authenticity, emotional resonance, and perceived usefulness of each vignette format.

**Results:**

This study demonstrates that manual and ChatGPT vignette creation methods are feasible approaches for analyzing and representing user-generated vlogs about living with chronic pain. Each method offers distinct utility: manual vignettes provide rich, nuanced narratives capturing emotional and social complexities, while ChatGPT-generated vignettes offer efficient, concise summaries with some loss of detail. Participant X’s reflections reinforced these findings.

**Conclusions:**

ChatGPT-generated vignettes, when combined with human review, offer the potential for an efficient and scalable approach to capturing the experiences of patients with chronic pain. These vignettes could support training, communication, and sharing of patient insights.

## Introduction

In recent years, the growing interest in patient-generated health data, personal data independently collected on various health aspects [1,2], has been fueled by advancements in technology. The integration of sensors in smartphones and wearable devices has empowered individuals to autonomously generate diverse health data, such as physical activity, heart rate, and sleep patterns [3-5]. This shift toward proactive well-being underscores a transformation in patient roles from passive recipients to active agents in their health care journey [6].

Alongside quantitative data, qualitative elements such as personal health video blogs (vlogs) have become an important platform for individuals to share their health experiences. While vlogs have been extensively used in citizen journalism [7], e-learning [8], product marketing [9], and personal communication [10], health vlogs represent a novel trend where individuals with medical conditions share their experiences. Peer support, especially for those with chronic illnesses, has demonstrated significant benefits through shared personal experiences [11]. Despite these benefits, limited research has explored methods to enhance patient connections within health vlogs.

There are different possible methods that could be used to assess qualitative health vlog data, such as autoethnography, phenomenology, grounded theory, and case reports [12]. Autoethnography allows for deep personal insights [13,14], while phenomenology focuses on understanding the essence of lived experiences [15,16]. However, they are not necessarily suited for vignette creation that can follow as an abstraction of these data.

Case reports have long served as powerful tools in medical education and narrative medicine, offering unique, nuanced, and individualized accounts that illuminate broader clinical or social issues [17,18]. When applied to consultation notes, patient experiences, or digital narratives such as health vlogs, particularly video recordings that capture specific or unique medical conditions, case-based methods provide a structured approach to contextualize these experiences. This framing can enhance clinical relevance and ground the narratives in the lived realities of individuals. In this study, a case report methodology was initially used to analyze a series of patient-submitted 1-minute recorded vlogs that focused on various situations, contexts, and conditions, including chronic pain. This involved a manual iterative process of watching, coding, and summarizing unstructured video data to extract central themes and construct patient-centered vignettes. These manually constructed vignettes served as the reference standard, against which AI-generated outputs were subsequently compared.

Narratives, integral to human communication, offer a structured way to document the passage of time, creating a coherent sequence with a beginning, middle, and end [19]. A narrative approach is particularly valuable in studying complex phenomena like chronic pain, aligning with multidisciplinary and holistic perspectives [20-22]. Moreover, narratives adapt to different topics and sensibilities, reflecting the storyteller’s evolving perspective.

Vignettes are short and carefully constructed depictions and offer a systematic representation of a person, object, or situation as a snapshot of specific situations or environments [23]. Vignettes, widely used across diverse fields such as social science, organizational research, psychology, business ethics, information studies, and nursing research, are narratives that provide insights into perceptions, beliefs, and attitudes [24,25]. While their definitions and applications vary, vignettes typically provide a text-based description of real scenarios that reflect lived experiences, social interactions, or complex decision-making situations. A standard vignette often includes a brief narrative written in the third person, presenting contextually rich details about a person, setting, or situation to prompt reflection, discussion, or analysis [26]. These narratives may incorporate elements such as dialogue, inner thoughts, or situational cues to enhance realism and engagement. This framework provides the structure used for the deductive comparison between the human- and ChatGPT-prepared vignettes in this paper. Although vignettes can be extended to include visual components such as images or video clips, this study focused exclusively on text-based formats based on the analysis of user-generated short, video-based narratives. The potential for multimodal or video-based vignettes is acknowledged and reserved for discussion in the future work section.

Currently, the process of preparing vignettes is time-consuming and labor-intensive, involving manual iterative sorting, coding, and summarization of raw qualitative data, often from multiple sources [27]. This process requires significant human effort and domain expertise, which can limit scalability, introduce subjectivity, and delay timely insights. Therefore, in practice, not all researchers engage in vignette development, which limits the broader use of qualitative data to generate vignettes or composite narratives.

In our research, video-based narratives are collected via a system called MyHealthMyRecord (MHMR) [28,29], and designed to enable multimedia expressions of experiences living with a chronic condition. It consists of a tablet-based video application that accepts short-duration audio or video user input and a server system for secure transfer, processing, and storage of video records. The user can organize video records along a linear timeline or by other organizing structures, such as theme, and then curate who can access various videos or organizing structures. For example, medical personnel could access a set of clips related to pain experiences, while a school accessibility specialist could access clips related to difficulties accessing school resources. It can function as a digital video scrapbook that documents and bundles a person’s experiences. The system can also produce summary visualizations such as bar and line graphs, word clouds, and text reports using AI-based analytics for changes over time, frequency of words or phrases used, and trends.

Prompt engineering refers to the deliberate crafting and refinement of input instructions provided to GenAI models to elicit accurate, contextually appropriate, and high-quality outputs [30]. In health care, especially when working with patient-generated content, the structure and specificity of prompts play a critical role in shaping the relevance and usability of AI-generated narratives. Carefully constructed prompts can reduce hallucinations, preserve clinical nuance, and enhance overall coherence, making them particularly valuable for converting complex data into meaningful summaries [31]. When researchers work with unstructured data such as audio or video recordings, prompt engineering can offer a way to tailor the behavior of large language models for clinical or research applications, including vignette development [32,33]. Therefore, prompt engineering is an essential methodological step in maximizing the reliability of GenAI for patient-centered communication tools.

GenAI tools, such as the current version ChatGPT-5, have demonstrated potential in summarizing and transforming large volumes of unstructured qualitative data [34]. In our research, we used prompt engineering to strategically design input instructions guiding the GenAI system to create relevant and representative vignettes from the same video dataset that was used to manually construct the equivalent vignettes. Prompts were refined through multiple rounds to ensure clinical coherence, preservation of narrative voice, and contextual accuracy. This iterative prompt design process was crucial in aligning GenAI outputs with the intended educational and communication goals.

This study compares the feasibility and utility of 2 different vignette creation methods, manual and ChatGPT-3.0, herein called ChatGPT, from user-generated vlogs about living with chronic pain. We examine how ChatGPT can offer a scalable and efficient alternative by rapidly generating coherent and contextually appropriate narratives from unstructured data, potentially reducing researcher or health care professional (HCP) burdens and accelerating the development of clinically relevant materials, while still requiring expert review to maintain validity and ensure conceptual rigor and authenticity. Specifically, we compare 3 vignettes that were created manually by an expert vignette producer and a version produced by ChatGPT for the same patient-generated data. We also describe the prompt engineering process that occurred for the ChatGPT-generated vignettes. Our research question is: how do manually prepared vignettes compare with AI-generated vignettes in terms of efficiency, effectiveness, and user relevance for patients living with chronic pain?

## Methods

### Overview

This section describes two different methods and steps of vignette creation using (1) a manual process and (2) an AI-generated using a member check approach [35] for coassessment and validation. This study proposes a comparative methodological workflow for vignette creation that integrates manual narrative construction and ChatGPT-assisted generation using identical patient-generated video transcripts, thereby potentially improving scalability and enabling methodological reflection on efficiency, consistency, and narrative fidelity. For both methods, the same user vlogs generated with the MHMR system were used, so the transcriptions were the same.

### Ethical Considerations

This study was approved by the Toronto Metropolitan University’s (formerly, Ryerson University) Human Research Ethics Board (Protocol # REB 2016‐150). The study followed all necessary procedures to ensure the confidentiality of the participant. Prior to the study, the participant co-designer completed and signed a written informed consent document. All data were deidentified prior to analysis and reporting to protect participant privacy. The participant was informed of their right to withdraw from the study at any time without consequence. All methods were carried out in accordance with relevant institutional guidelines and regulations. The use of generative AI to transform patient-generated narratives also introduces potential risks of narrative distortion and interpretive bias, which were mitigated through iterative human review and validation against the source transcripts. Written informed consent was obtained from the individuals for the publication of any potentially identifiable images or data included in this paper.

### Case Background and Data Collection

The user co-designer (Participant X), who recently experienced a sudden chronic illness, was tasked with using the MHMR application for 3 months. The purpose was twofold: to assist in refining the interface and server functions of the system, and to assess the usefulness of the platform from Participant X’s perspective. Participant X began using the system during a 3-week business and leisure trip and continued afterward for 11 weeks while attending school. During the travel phase, Participant X recorded 18 videos, with an average length of 1 minute (ranging from 31 to 70 seconds). After returning, Participant X recorded an additional 29 videos, with an average length of 40 seconds (ranging from 17 to 148 seconds).

Participant X was selected as the user co-designer due to the nature of the challenges posed by the sudden onset of illness or disability that was not diagnosed, which presented an interesting and unique health case. Participant X was encouraged to document their experiences of physical pain, challenges, successes in daily tasks, frustrations, and any other significant events through unscripted, unscheduled videos. A thematic analysis [28] was carried out with a panel of 2 content researchers and senior experts in qualitative studies and digital technologies for natural language processing and health sciences. In total, 8 main themes were identified and further categorized into 3 domains: person-facing, accessibility experiences, and system-facing. These domains corresponded to the 3 streams of Participant X’s life: as a university student, as an international traveler, and as a patient living with chronic pain. For a detailed reporting on the methodology and results of the thematic analysis, see Lakha et al [28]. It was decided to produce vignettes reflecting the 3 streams of Participant X’s life.

### Data Sources and Vignette Creation

Data sources included 3 videos for each topic, a text transcription for each video generated by a researcher (Textbox 1), and written reflection narratives on critical incidents portrayed in each video generated by a study researcher. This yielded a total of 9 video clips, 9 narrative data segments, and 3 summaries that were used to generate 3 vignettes for the specific Participant X profile.

Textbox 1. **Transcription of videos created by Participant X.**

**Vignette 1: university student (Participant X)**

Scenario: Participant X attends university and documents experiences using the MyHealthMyRecord (MHMR) tool.

Verbatim of recorded videos

- “So it was a pretty interesting event in yesterday’s class, in the morning, so my professor was talking, talking, explaining, and this was in the beginning of class, so I respectfully had my hand up for a while, then he’s like you have your hand up for a while, and there’s nothing to be discussed, going over stuff and I m just like yeah ... ‘can you just speak louder’ and, he said ‘I can’t that the loudest I can speak, and I said ‘I cannot hear you,’ so he said ‘come to the front’ pretty much, ... I was shocked that he said that, and I said ‘but I can’t,’ and he said ‘well I guess we’re stuck we can’t do much about it’ and ‘I’m like ‘I guess so’.”
- “Oh my gosh, University X needs to fix those automatic doors that aren’t working. First of all, they’re so heavy so I can’t push them, and the people are so inconsiderate, they see me struggling, and I’m trying to push it, and they still don’t come by to help me. I don’t know which world they’re living in but that’s pretty rude I would say, but I guess they just don’t care, got to deal with it.”
- “I wanted to talk about my evening class where we are talking about the wheelchair manufacturer industry, and my professor kept on putting me on the spot, which was not nice; we were talking about something about wheelchairs he could just generalize and make statements he doesn’t have to put me on the spot like what does patient X feel like ... its just that I’m in the wheelchair doesn’t give him the license to sort of put me on the spotlight, and it was very uncomfortable and not nice. I didn’t get a chance to speak to him, but for sure, I’m going to follow up with him by email for this.”

**Vignette 2: traveler (Participant X)**

Scenario: Participant X travels frequently for personal and academic reasons, documenting his experiences with the MHMR tool.

Verbatim of recorded videos

- “Today is Thursday; the learning journey was pretty cool, and we woke up early and went to a local school in Hanoi, Vietnam ... We got a nice lunch, and it was very accommodating. But the school itself is not very accessible, about 10 people had to pick up my wheelchair on the main four steps, and I stayed in the main area. I did not go to the washroom but went to wash my hands, and it was very tight. It’s not a very accessible place.”
- “The weather in Denver is horrible in the sense that it’s the same as Toronto; it snowed a lot, and no one cleaned it up. Snow on the sidewalk, the road everywhere, so hard to walk I was slipping and off balance; I didn’t expect that; I expected the city to be bigger than what was plus cleaner than it was because it was the main downtown, so I was surprised with that.”
- “So Sydney has been treating me great, I love it; it’s a beautiful city, very accessible, very accommodating, but too many hills, and it’s tough getting around in that sense. People are very helpful, and accessible, being so accommodating, there’s these accessible taxis, they look like funeral cars, pretty disturbing, but they are accessible in that sense, it’s very nice.”

**Vignette 3: patient with pain (Participant X)**

Scenario: Participant X tracked instances and the frequency of personal struggles with pain, documenting them using the MHMR tool.

Verbatim of recorded videos

- “It was not as warm as I thought; only one day it was 84F; otherwise it was 40-50F, which is chilly, and it adds on my pain and ability to walk.”
- “Today was the first time (Physiotherapy) had at a 9 am session ... worst decision ever, my body was so stiff, and I got drained out, it was too difficult for me.”
- “I’m not been getting enough sleep for the past two or three days and, on top, have pain in the knees and ankles sore ... Hopefully, things will get better, and exams and assignments have to end so that I will rest up. Looking forward to it.”

The study researcher has over 15 years of experience developing case reports and vignettes from patient data in collaboration with a clinical principal investigator, drawing on consultation notes, administrative records, and psychometric assessments [36,37]. The existing constructs in this framework, outlined by Mailis-Gagnon et al [36] and Lakha and Mailis [37], have been used for several years; therefore, we considered their external validity to be well established. This existing framework was then used for the creation of the human-generated vignettes and for the AI prompting for ChatGPT-generated vignettes, as well as for the deductive comparison between the 2 vignettes. This work involved synthesizing clinical and psychosocial data into structured, person-centered narratives that reflected key symptoms, diagnoses, and contextual factors. In this study, we applied the same approach to analyze and summarize video-based patient narratives, translating them into meaningful vignettes. In addition, the vignettes were reviewed and validated by senior researchers (PP and DIF), along with Participant X, through participant feedback, participant member and expert review, and iterative refinement with human oversight, ensuring thematic fidelity, realism, and alignment with source narratives.

### Manual Vignette Creation Process

Manual vignette creation was carried out by one researcher to generate concise, text-based narratives that captured the essential themes and emotional tone of the videos and narrative data across the 3 streams of Participant X’s life. The study researcher followed a detailed, systematic approach to ensure the accuracy and relevance of the content. The researcher transcribed the 9 video recordings, a critical first step to capture all verbal details accurately. This process took about 5 hours. To gain a deeper understanding of Participant X’s thought process, the researcher then spent 3 hours generating and clarifying narratives that reflected Participant X’s perspective. Each narrative was approximately 70‐200 words in length and provided a stream-focused and context-rich account of the participant’s experience. These resources were analyzed to extract key concepts, events, and critical moments that shaped Participant X’s experience. The researcher manually synthesized the extracted information, ensuring the content’s accuracy by reviewing and cross-referencing the data sources, specifically, comparing the transcripts with the original video recordings and Participant X’s previous reflections to confirm consistency in concepts, tone, and contextual details. This process was done to capture key moments and create a cohesive narrative. This step took an additional 4 hours. Finally, the researcher used an iterative process to refine and improve the vignette summary. The purpose of this refinement was to ensure that the vignette accurately reflected Participant X’s lived experience, captured the emotional nuances of the original data, and maintained narrative coherence. The researcher considered the vignette complete when no new changes were needed upon rereading and when the narrative consistently aligned with the key concepts identified across the transcript and video. This final iterative process took about 5 hours.

The total time for the manual vignette creation process, excluding the transcription, was approximately 8 hours. Including the transcription step, the total time spent was 13 hours. Learning time was not included in this estimate as the researcher had over 20 years of experience creating manual vignettes in the medical discipline.

### GenAI-Assisted Vignette Creation

We used OpenAI’s ChatGPT-3.0 (first and free version) as the GenAI tool for the vignette creation. ChatGPT will be used in this section as the representative example of GenAI. This was the researchers’ first experience with using ChatGPT to produce vignettes. The process involved using the same transcription from the raw video data that was used for the manual vignette method, ensuring the preservation of participant privacy and confidentiality. Each text transcript was uploaded to ChatGPT and, using an iterative prompt engineering process [38,39], was asked to generate a narrative summary. The prompt engineering method required iterative refinement to ensure that the ChatGPT summaries aligned with the researchers’ expectations. The researchers spent approximately 45 minutes refining the prompts, aiming to elicit accurate and coherent summaries. Prompt engineering was conducted as a systematic analytic process. In total, 5 prompt iterations were tested and refined to ensure that the ChatGPT summaries captured the intended narrative content, emotional tone, and contextual meaning. The final prompt was selected based on the ability to maximize coherence, thematic fidelity, and alignment with source narratives, with iterative adjustments documented to maintain accuracy and minimize misrepresentation. As part of the initial prompt engineering approach, ChatGPT was asked to “provide the narrative of each transcript.” This served as a starting point that reflected the intention to explore how ChatGPT could support the summarization of qualitative data. However, the initial outputs were often vague and did not capture the expected nuances. The prompts were subsequently refined through multiple iterations, incorporating more specific terms such as “summary,” “explain,” “situation,” “expressing,” and “this” to guide ChatGPT’s processing of the content. Over approximately 45 minutes, the different prompt variations were systematically tested, and ChatGPT’s responses after each attempt were carefully reviewed with respect to the expected nuances and analytic objectives. The final prompt that consistently yielded coherent and contextually relevant summaries was: “Provide a summary of what the participants are expressing in this transcript?”

Evaluation of prompt performance was conducted through iterative researcher judgment rather than formal quantitative or coded assessment, guided by the study’s exploratory and feasibility-oriented design. Prompt success was primarily determined by the degree to which outputs reflected transcript coherence, contextual fidelity, and narrative completeness relative to the source data.

This iterative process involved not only refining the technical elements of prompt development but also incorporating the researchers’ interpretive judgment and evolving understanding of the data to guide interactions with the AI tool. It illustrates a partial autoethnographic engagement, as she drew on her own positionality, judgment, and evolving understanding of the data to interact meaningfully with the AI tool during the analysis.

Once these summaries were generated, they were reviewed for accuracy and completeness, compiled into the 3 thematic topics with 3 video transcripts each, and then resubmitted to ChatGPT to produce a vignette.

Using iterative prompt engineering, the researchers generated a final question to produce the vignettes. The researchers iterated the prompt 4 times to ensure that the final summary captured the essence of each individual incident. This process also took about 45 minutes. The final prompt for the ChatGPT-created vignettes was:

*Summarize the three incidents shared by the participant regarding their experiences at the place (e.g. University, Traveling). Identify the key events described in each incident and provide an overarching statement that captures the essence of the participant’s concerns or experiences, highlighting any common themes or issues raised across the incidents.*

The entire ChatGPT vignette production, without transcription, took a total of 3 hours.

Following the manual and ChatGPT vignette creation process, the 3 vignettes produced using each method were reviewed by Participant X to determine differences and usefulness. Participant X took about 1 hour to review all vignettes.

## Results

The vignettes produced in this study, through manual and ChatGPT methods, were intended to capture key aspects of Participant X’s lived experience during their 3-month engagement with the MHMR application, organized by 3 topics, university student, traveler, and patient with pain, as identified in a thematic analysis of the original data [28]. Through the iterative vignette creation process, both methods provided similar and unique insights into Participant X’s challenges and triumphs while navigating the impact of chronic illness on daily life.

### Vignette Creation

An analysis of the manually created vignettes demonstrated a consistent conceptual presence across the narratives, with repeated references to the challenges of maintaining a normal life while managing a chronic condition. These vignettes conveyed a sense of Participant X’s evolving adaptation to their illness, highlighting the emotional toll and the strategies used to regain a sense of control over their daily routines. Textboxes 2-4 present the vignettes that were created.

Textbox 2. **Vignette 1—individual profile: university student, illustrating the experiences and challenges faced by Participant X as a domestic student enrolled in a degree program at the university.**

**Manually generated narratives for each video**

- “The participant was attending the class from the back seat as he could not move forward due to her wheelchair and classroom structure, so he asked a professor to speak loudly, but to his astonishment, the professor asked her to come to the front without considering his situation.”
- “The participant faced the issue of automatic doors that were not working for a while and they were so heavy that she couldn’t push and people around him were not considerate. She faced accessibility challenges at the campus and expressed frustrations with inaccessible physical environments, especially those that made him reliant on others for assistance.”
- “Because a participant used a wheelchair, individuals put her on the spot to discuss the issue of products and services used by people with disabilities. He will follow-up with professor.”

**Summary of 3 videos**

- Participant X attended classes regularly at the university, and every day he/she encountered issues of ethical and inclusive environments at the institution.

**ChatGPT-generated narratives for each video**

- “The student recounts an incident from yesterday’s class where the professor was explaining something at the beginning. Despite raising their hand to contribute, the professor dismissed it, claiming there was nothing to discuss. When the student asked the professor to speak louder, the professor said it was not possible. The professor then suggested the student move to the front, which the student couldn’t do. This left the student shocked, and despite expressing their inability to comply, the professor indicated they were stuck and couldn’t do much about it.”
- “The participant is expressing frustration with the malfunctioning automatic doors at University X. They highlight the doors’ weight, making it difficult to push, and criticizes others for not offering assistance despite witnessing their struggle. This lack of assistance is seen as inconsiderate and rude by the participant, who feels they are left to deal with the problem on their own.”
- “The participant is discussing their experience in an evening class focused on the wheelchair manufacturer industry. They express discomfort with their professor repeatedly singling them out during discussions, specifically asking them personal questions like ‘What does PX feel like?’ The participant feels being in a wheelchair shouldn’t warrant such spotlighting. They intend to address the issue with the professor via email.”

**Summary of 3 videos**

- The participant recounts three recent experiences: feeling disregarded in class discussions, encountering difficulty with malfunctioning automatic doors on campus, and experiencing discomfort when singled out in discussions about wheelchair manufacturing. These incidents underscore the need to promote inclusivity, accessibility, and sensitivity within the university community.

Textbox 3. **Vignette 2—individual profile: international traveler with accessibility needs, exploring the physical and emotional challenges encountered by Participant X, a domestic student who requires accessibility services while traveling.**

**Manually generated narratives for each video**

- “The participant shares their personal experience of a long but pleasant journey to school in Hanoi, Vietnam, on a weekday. They describe having lunch in a light mood and how the institution managed to accommodate the situation. The participant also recounts how the academic institution was not accessible, as several people required a wheelchair to move from the ground to the main floor, which was only accessible by four steps. This led them to stay in the main area to avoid causing inconvenience to others. The participant also explained how washrooms were not wheelchair-accessible, and there was limited space, even for washing hands.”
- “A participant compares the weather in Denver to that of Toronto, finding it equally horrific and similar. They describe heavy snowfall with no one clearing the roads or sidewalks, and due to their situation, they were unable to maintain balance and slipped. This was not what they expected, as they had assumed Denver, being a big city, would be cleaner, especially in the downtown area. They were surprised by the situation.”
- “A participant expressed satisfaction with how Sydney treated them, appreciating the city’s accessibility and accommodation. However, they noted that the city’s hilly terrain made it difficult for them to navigate. The participant found the people in Sydney helpful and accommodating. They also described the accessible taxis, which resembled funeral cars. While this was disturbing to them, they still appreciated that the taxis were accessible.”

**Summary of 3 videos**

- Participant X frequently travels for both personal and academic reasons and shares his experiences along the way regarding the accessibility and accommodation of different places, and how these experiences impact him. He also discusses the frustrations and disappointments he faces during his travels, particularly how big cities lack important accessibility services.

**ChatGPT-generated narratives for each video**

- “The participant shares their experience of a day in Hanoi, Vietnam, describing their learning journey as enjoyable. They woke up early, visited a local school, and had a nice and accommodating lunch. However, they express frustration with the school’s lack of accessibility, mentioning that around 10 people had to carry their wheelchair up four steps to the main floor. They also note the limited space in the washroom area, which made it difficult to wash their hands. Overall, the participant highlights that the school is not very accessible.”
- “The participant expresses disappointment with the weather in Denver, comparing it to Toronto. They mention heavy snowfall that was not cleared, making it difficult to walk and causing them to slip and lose balance. The participant did not expect this situation, as they anticipated a cleaner and more organized city, especially in the main downtown area, and they were surprised by the lack of maintenance.”
- “The participant expresses their overall positive experience in Sydney, describing the city as beautiful, accessible, and accommodating. However, they mention the challenge of navigating the city’s many hills. They also appreciate the helpful and accommodating people in Sydney. While they find the accessible taxis, which resemble funeral cars, somewhat disturbing, they acknowledge that the taxis are indeed accessible and appreciate their convenience.”

**Summary of 3 videos**

- The participant shares three experiences related to accessibility during their travels. In Hanoi, they found the local school accommodating in some aspects, but struggled with wheelchair inaccessibility, requiring others to lift their chair up steps and facing tight spaces in the washroom area. In Denver, they were disappointed by the lack of snow removal, which made walking difficult, especially in the downtown area where they expected better accessibility. In Sydney, the participant praised the city’s accessibility and helpful people but struggled with navigating hilly terrain, despite the availability of accessible taxis. The common theme across these incidents is the participant’s mixed emotions of appreciation and frustration due to accessibility challenges in different locations.

Textbox 4. **Vignette 3—individual profile: university student managing chronic pain, depicting the experiences and challenges of Participant X as a domestic student, balancing academic responsibilities with chronic pain management, including the impact of weather, physiotherapy, and physical fatigue.**

**Manually generated narratives for each video**

- “A participant identified and tracked instances related to the weather, reflecting on the chilly conditions and describing the physical pain experienced during the day due to the cold weather, which restricted their walking ability.”
- “An individual scheduled their first physiotherapy session in the morning and was frustrated and disappointed with their decision, as mornings were difficult due to pain. It was hard for PX, and they felt completely exhausted afterward.”
- “A participant described struggling with restless sleep for the past few days and also suffering from soreness in their ankles and knees, which reflects their frustration. However, the participant is hopeful that their pain will improve in the future once they finish their assignment and are able to rest. PX is looking forward to a break from both pain and other stresses.”

**Summary of 3 videos**

- Participant X identified and tracked instances and the frequency of pain, exhaustion, stress, irritation, and frustration, reflecting on when these emotions occur and examining trends in sustained negative emotional states. This included experiences such as physical pain from cold weather affecting mobility, frustration from scheduling physiotherapy in the morning while dealing with pain, and struggling with restless sleep and soreness in the ankles and knees. Despite these challenges, Participant X remains hopeful for future improvement.

**ChatGPT-generated narratives for each video**

- “The participant is expressing that the weather was colder than expected, with temperatures mostly between 40-50°F, except for one day at 84°F. They note that the chilly conditions aggravated their pain and affected their ability to walk.”
- “The participant is expressing regret about scheduling their first physiotherapy session at 9 a.m., describing it as a difficult decision. They felt physically stiff and drained afterward, finding the experience too challenging.”
- “The participant is expressing frustration with not getting enough sleep for the past few days and dealing with soreness in their knees and ankles. Despite these challenges, they are hopeful that things will improve once exams and assignments are over, and they look forward to getting some rest.”

**Summary of 3 videos**

- The participant recounts three recent experiences: the impact of cold weather on their mobility and pain, the regret of scheduling a challenging 9 a.m. physiotherapy session, and struggling with poor sleep and physical soreness. These incidents highlight the participant’s ongoing physical challenges, the difficulty of balancing health with academic responsibilities, and the hope for relief once academic pressures ease.

### ChatGPT Vignette Creation

The ChatGPT vignette creation process successfully generated summaries that captured key events and overarching concepts from the video transcripts. The iterative prompt engineering process enabled the researcher to refine ChatGPT summaries, ensuring alignment with the life stream focus of the study.

The ChatGPT-generated vignettes reflected similar concepts to those found in the manually created vignettes, such as Participant X’s emotional responses to health setbacks and their interactions with others. However, the ChatGPT-generated vignettes were generally more concise and sometimes omitted important details related to emotional expressions or complex social dynamics that were included in the manually created versions. While ChatGPT identified major incidents and synthesized them into cohesive narratives, Participant X perceived that these vignettes occasionally lacked the depth of personalization achieved through manual creation (Textboxes 2-4).

A comparative review of both sets of vignettes, based on Participant X’s reflections, suggested that the manual vignettes were perceived as capturing richer, more contextually detailed accounts with personal nuances. In contrast, the ChatGPT-generated vignettes were perceived as faster, and more streamlined, but with some reduction in emotional and social complexity.

### Comparative Analysis

In comparing the outcomes of manual and ChatGPT vignette creation methods, several points of distinction emerged. To support a structured comparison aligned with the study’s exploratory aim, the analysis focused on key qualitative dimensions including efficiency, narrative coherence, emotional nuance, and contextual completeness, as reflected in the vignette outputs and Participant X’s interpretive feedback. The most important difference between the 2 was time and efficiency. After the initial vignette creation with prompt engineering, which took several hours, all subsequent vignettes generated using ChatGPT were faster, with each vignette taking roughly 10 minutes to generate, compared to about an hour for each manual vignette. This speed is particularly relevant in larger volume applications where time efficiency is crucial. While ChatGPT-generated vignettes were accurate and concise, manual vignettes provided deeper insights into Participant X’s emotional and cognitive responses, offering more detailed contextualization of incidents and challenges. Both methods captured the major events from the video content and transcriptions and produced consistently nuanced vignettes reflecting Participant X’s experiences. However, Participant X perceived that, in some instances, manual creation captured more precise emotional tones and reflective thought processes. These findings suggest that while ChatGPT can be a useful tool for rapidly generating vignettes, the manual creation process remains essential for producing deeply nuanced narratives that accurately reflect the complexity of personal experiences. These dimensions were not formalized as a coding framework; however, they provided a consistent basis for comparative interpretation across both methods within the feasibility-focused scope of this study.

### Participant X’s Reflection and Commentary on Vignette Creation

Participant X provided reflections on the vignettes, describing perceived differences in emotional depth and narrative style between manual and ChatGPT approaches (Textbox 5).

Textbox 5. **Participant X’s reflections on manual and AI-generated vignette creation.**

- Reviewing the researcher’s summary and vignettes helped validate my emotions and the intangible aspects of my experience, like feelings. It captured the key events while also weaving in the emotional aspect, which humanized the process and showed compassion and empathy for what the patient or user would be going through. As a user, this feels validating for my personal health, mental health, and emotional well-being. Interestingly, the analysis showed patterns in my mood around certain events. I had not noticed before how my stress levels were linked to certain triggers in my environment.
- The ChatGPT summaries concisely capture the overarching themes in a factual manner. Where emotions are mentioned, it is more of a verbatim capture of what I explicitly said, like frustration or fatigue. This approach feels more dehumanized and focuses on key points, which are important for people who need to understand the situation, like health care providers and those in the circle of care. They need facts to understand what’s happening without emotions clouding their judgment. Additionally, while ChatGPT suggests that I am experiencing an episode of pain, I still feel pain daily, which makes me think that perhaps it does not account for my emotional response to the pain.
- Both approaches have their strengths, but the choice depends on the need. When a therapist and/or someone in the circle of care is working to address emotional well-being, it is valuable to capture the emotional aspect. In contrast, the fact-based approach is more important for health care providers and service providers who need a quick, clear understanding to provide timely and appropriate care. While the latter may lack emotional depth, in the future, this could be addressed by adding prompts to ChatGPT to focus on capturing emotions more specifically. Overall, I think this process has helped me understand my condition more deeply. It is empowering to have my experiences analyzed in such a structured way, and I feel more in control of my health.
- As a patient or user, this technology is also helpful for those who struggle to put their thoughts and feelings into words and cohesively communicate their experiences. ChatGPT could assist in conveying this, and users could review the output to ensure that it accurately reflects the extent of their condition.

## Discussion

### Summary of Key Findings

This study aimed to explore two approaches to vignette creation: (1) manual and (2) ChatGPT (an example of GenAI), to summarize and present Participant X’s experiences in managing a chronic condition. The objectives for the vignettes were to illustrate the physical and emotional experiences and challenges faced by Participant X in each of the 3 life streams: as a university student, as an international traveler, and as a patient with chronic pain. Both approaches provided similar vignettes of Participant X’s lived experience, highlighting how they can serve as powerful tools for communicating complex health-related challenges in text-based summary forms. The ability of an app-based tool to convert raw patient experiences into structured vignettes represents an innovative step in patient-centered storytelling, offering scalable solutions for health care communication.

Vignettes serve as a bridge between raw personal experiences and researcher- or HCP-structured narratives that can inform reflection and analysis of patient-reported experiences with health care services, their benefits, limitations, and areas that need improvement. They articulate patient needs, struggles, and coping mechanisms in a manner that is both accessible and actionable for health care providers, caregivers, and support networks. By presenting real-life challenges in a structured format, vignettes help decision-makers understand the realities of daily life with chronic conditions and pain, making it easier to develop personalized, patient-centered care strategies.

ChatGPT vignette creation is particularly advantageous due to its efficiency, reduced time commitment, and ability to standardize case studies that could be used for clinical training and policy development [40]. Within this single-case exploratory study, Participant X’s reflections suggested that ChatGPT vignette creation was perceived as particularly advantageous due to its efficiency and reduced time commitment. In contrast, manual vignette creation typically requires substantial domain expertise and a deep understanding of the individual’s broader context to produce richly detailed and emotionally nuanced narratives, which may limit scalability in multicase or time-sensitive applications. Bensing [41] suggests that structured narratives help providers better understand patient struggles, leading to improved care strategies tailored to individual needs; thus, having large quantities representing many unique cases could be beneficial and cost-effective. From Participant X’s perspective, vignettes also appeared to support patient communication and self-reflection within their circle of care. In addition to informing clinical practice, vignettes empower patients to articulate their needs within their circle of care. Chronic pain and other long-term conditions often require ongoing communication with multiple stakeholders, including caregivers, employers, and educators. By crafting vignettes that depict specific struggles and achievements, individuals can self-reflect on their experiences and situations as well as advocate to others for the accommodation and support they require, with the evidence provided from the vignettes. Vignettes can serve as accessible, structured narratives that help individuals explain their lived experiences as summaries rather than from raw, unfiltered data (in written, audio, or video forms) [42]. The ability to generate vignettes through ChatGPT also democratizes the process, enabling patients to document and share their experiences without requiring extensive writing or data analysis skills.

This project adopted a first-person, phenomenological approach, centering Participant X’s voice in the storytelling process. Unlike traditional case studies that rely on external interpretations by clinicians and researchers, these vignettes preserve the authenticity of Participant X’s lived experience through their reflections [43]. This approach was intended to preserve key incidents along with emotional and cognitive dimensions of Participant X’s lived experience. ChatGPT-generated vignettes must continue to be refined to ensure that they capture the expected nuances to avoid depersonalization or hallucinations in the vignette. The prompt was effective because it aligned with how large language models and GenAI like ChatGPT process natural language using semantic and contextual cues. The development of the prompt phrasing guided ChatGPT to focus on intent, contextual structure, and semantic meaning rather than using isolated keywords as recommended by Moreau et al [44] and Topol [45]. This was especially relevant, given that the data consisted of person-generated narratives, typically drawn from real-life scenarios, which often include fragmented, emotional, or conversational language. Each word in the final prompt thus guided the model to recognize intention, tone, and relational meaning embedded in the dialogue. This likely contributed to the coherence and contextual relevance of the generated summaries, illustrating how prompt design can serve as a bridge between computational processing and qualitative data in this exploratory context.

### Implications for Future Research and Practice

The findings suggest that manual and GenAI, exemplified in this case by ChatGPT, vignette creation have distinct advantages. Manual vignettes capture deeper emotional and cognitive insights, while ChatGPT methods offer efficiency and scalability. Future research could explore hybrid models that combine ChatGPT’s efficiency with human expertise to refine and personalize vignettes in order to exploit the advantages of each with a single process becoming GenAI-assisted, rather than exclusive, vignette creation process.

ChatGPT-assisted vignette creation potentially provides a cost-effective, scalable way to depict chronic pain experiences and assess care quality. As health care systems integrate digital tools, the ability to generate structured, patient-centered narratives quickly will be increasingly valuable [45,46]. Future research should enhance GenAI-assisted vignettes by optimizing speech-to-text transcription and balancing video quantity with quality to improve accuracy and depth for patient advocacy, clinical training, and policy development. In addition, understanding how HCPs can use individualized vignettes for patient or HCP interactions and communication could assist in designing or honing a set of prompts for specific purposes and needs.

Vignettes also play a key role in training HCPs by presenting real-world, context-rich scenarios that can enhance empathy, cultural sensitivity, and clinical decision-making [47]. By exposing medical trainees to diverse and unique patient narratives, vignettes can support the development of critical thinking and reflective practice in complex and nuanced situations [48,49]. The next step in this research could be to evaluate the advantages and disadvantages of ChatGPT-assisted vignettes in training HCPs.

In addition to their educational applications, vignettes have the potential to be integrated into digital health tools such as patient portals or mobile apps to help patients capture and communicate their signs and symptoms. This approach may offer a more structured way for individuals to share their lived experiences, thereby supporting personalized care and enhancing communication between patients and HCPs. We recommend further exploration of educational and digital applications of vignettes to support inclusive and patient-centered approaches in health care.

This study had a number of limitations. First, ChatGPT-3.0 is used, and the evaluation is based on the outputs generated as of April 2024. Because the accuracy of these technologies may change with future updates, regular assessments are necessary. Additionally, while ChatGPT-based vignettes enhance efficiency, they may fail to capture the depth of emotions, context, and subtle personal experiences that manual vignettes can provide. Other GenAI tools may produce different results and should be investigated in future research. Additional prompt engineering design is required to ensure that vignette objectives can be met. In addition, a single prompt may be insufficient to cover all potential needs. As such, research is required to determine how specific prompts can be structured to meet various patient or user needs.

The accuracy of AI-generated, specifically ChatGPT, vignettes also depends on high-quality speech-to-text transcription, and errors in transcription could lead to misinterpretation or loss of critical details. Additionally, this study relied on a single participant (Participant X) as the creator of the original content performing a member check [35], consistent with its exploratory, phenomenological design [50]. As a result, findings primarily illustrate feasibility, utility, and member check validation, and should be interpreted with caution regarding broader applicability to other patient populations or health care settings. Accordingly, interpretations of the usefulness of AI-generated vignettes for health care communication and training should be considered exploratory and illustrative within a single-case design. Furthermore, using ChatGPT or other GenAI tools for vignette creation raises concerns about data privacy, consent, ethical considerations regarding transcript contributions to learning models without consent and lack of knowledge, and the potential risk of misrepresenting patient experiences, making it essential to ensure confidentiality and transparency of how ChatGPT uses data such as transcripts while preserving authenticity. The researcher who worked with manual vignettes had many years of experience in generating case studies for patients with chronic pain, which is an important factor to consider when comparing the 2 approaches, as accounting for learning time was unnecessary. This expertise may introduce comparator bias, potentially enhancing narrative richness and emotional nuance relative to ChatGPT-generated vignettes, despite the safeguards implemented through multiple data sources and iterative validation. Therefore, researchers need proper training and a thorough understanding of patients’ distinct experiences to generate and/or evaluate meaningful and accurate vignettes.

The findings may not be applicable across different health care settings or patient populations, as factors such as cultural differences, literacy levels, and digital access can impact the effectiveness of ChatGPT-assisted vignettes. ChatGPT vignettes also rely on well-structured prompts and quality video transcripts, meaning poorly curated inputs could result in oversimplified narratives that lack depth and relevance. Additionally, while using ChatGPT accelerates vignette creation, human intervention remains necessary to refine narratives, correct inaccuracies, and ensure contextual appropriateness, limiting the possibility of full automation. These limitations highlight areas for further research and refinement to enhance the reliability and applicability of ChatGPT-assisted vignettes in health care communication.

The vignettes in this study were only text-based; no other media were used. However, there is a growing preference for video-based vignettes, as opposed to written ones, because video is seen to better present the nuances of real-life experiences and individual behavior, especially in health care contexts [51]. This study demonstrates that even early stage transformer-based GenAI applications like the one used here (ChatGPT-3.0) can be effectively used in the workflow process of generating multimedia vignettes from the raw video vlogs produced in MHMR, but patient confidentiality, privacy, and content ownership must be considered and explored in this context [52]. Finally, the vignettes created manually and by ChatGPT were not evaluated by HCPs, limiting insight into their practical usefulness. Future studies will include HCP assessment to enhance clinical relevance.

### Conclusions

The novel contribution of this paper is methodological, as it outlines a comparison between 2 different methods for generating vignettes, human and GenAI. This approach integrates patient-generated video narratives, manual vignette construction, GenAI-assisted summarization implemented with ChatGPT-3.0, and member checking to propose and test a participatory workflow for vignette development. Vignettes can serve as a powerful medium for illustrating individual struggles with chronic pain, offering qualitative insights and practical applications in clinical care. GenAI-assisted vignette creation may streamline this process, making it an innovative tool for HCPs, their assistants and service providers, patients, and policymakers alike. As technology continues to evolve, integrating high-quality GenAI-produced vignettes into health care settings may enable more personalized, effective, and empathetic care solutions.

## Abbreviations

HCP: health care professional
MHMR: MyHealthMyRecord
vlog: video blog
